# Enhanced biocidal activity of Au nanoparticles synthesized in one pot using 2, 4-dihydroxybenzene carbodithioic acid as a reducing and stabilizing agent

**DOI:** 10.1186/1477-3155-11-13

**Published:** 2013-04-22

**Authors:** Syeda Sohaila Naz, Nazar Ul Islam, Muhammad Raza Shah, Syed Sartaj Alam, Zafar Iqbal, Massimo Bertino, Louis Franzel, Afifa Ahmed

**Affiliations:** 1 Institute of Chemical Sciences, University of Peshawar, Peshawar 25120, Pakistan; 2 H.E.J. Research Institute of Chemistry, International Centre for Chemical and Biological Sciences, University of Karachi, Karachi 75270, Pakistan; 3 Department of plant pathology, Khyber Pakhtunkhwa Agricultural University Peshawar, Peshawar 25120, Pakistan; 4 Department of Physics, Virginia Commonwealth University, Richmond, VA 28234, USA; 5 Department of Chemistry, Sarhad University of Science and Technology, Peshawar Campus, Peshawar, Pakistan

**Keywords:** 2,4-Dihdroxybenzene carbodithioic acid, Gold nanoparticles, *Myzus persicae*, *Aspergillus niger*

## Abstract

**Background:**

The conjugation of gold nanoparticles with biocides such as natural products, oligosaccharides, DNA, proteins has attracted great attention of scientists recently. Gold NPs covered with biologically important molecules showed significant enhancement in biological activity in comparison with the activity of the free biocides. However, these reports are not very systematic and do not allow to draw definitive conclusions. We therefore embarked in a systematic study related to the synthesis and characterization of biocidal activities of Au nanoparticles conjugated to a wide variety of synthetic and natural biomolecules. In this specific report, we investigated the activity of a synthetic biocide, 2-4, Dihydroxybenzene carbodithioic acid (DHT).

**Results:**

Au nanoparticles (NP) with a mean size of about 20 nm were synthesized and functionalized in one pot with the help of biocide 2,4-Dihydroxybenzene carbodithioic acid (DHT) to reduce HAuCl4 in aqueous solution. Conjugation of DHT with gold was confirmed by FT-IR and the amount of DHT conjugated to the Au nanoparticles was found to be 7% by weight by measuring the concentration of DHT in the supernatant after centrifugation of the Au NPs. To ascertain the potential for in vivo applications, the stability of the suspensions was investigated as a function of pH, temperature and salt concentration. Antibacterial, antifungal, insecticidal and cytotoxic activities of the Au-DHT conjugates were compared with those of pure DHT and of commercially available biocides. In all cases, the biocidal activity of the Au-DHT conjugates was comparable to that of commercial products and of DHT.

**Conclusions:**

Since the DHT concentration in the Au-DHT conjugates was only about 7%, our results indicate that conjugation to the Au NPs boosts the biocidal activity of DHT by about 14 times. The suspensions were found to be stable for several days at temperatures of up to 100°C, salt concentrations up to 4 mol/L and a pH range of 2-13.

## Background

In nanotechnology, considerable effort is being devoted to the development of nano-bioconjugates for drug delivery, for photothermal therapy or for imaging purposes [1]. Nanoparticles and nanoparticle conjugates are also being considered as drugs of their own, which is not surprising since the biocidal activities of Ag and Cu have been known for centuries [2]. So, for example, Ag nanoparticles are being incorporated into textiles and are marketed globally under the SmartSilver™ brand [3]. More recently, reports have appeared which indicate that Au nanoparticles enhance the biocidal activity of biomolecules conjugated to them [4]. However, these reports are not very systematic and do not allow to draw definitive conclusions. We therefore embarked in a systematic study of synthesis and characterization of antibacterial, antifungal, insecticidal and cytotoxic properties of Au nanoparticles conjugated to a wide variety of natural and synthetic biomolecules. In this specific communication, we investigated the activity of a synthetic biocide, 2-4, dihydroxybenzene carbodithioic acid (DHT). The molecule was synthesized in our laboratory [5] and was considered attractive for several reasons. The presence of a thiol group ensured that DHT would conjugate to Au without requiring any post-synthesis processing. In addition, DHT, like most thiols is also a mild reducing agent which allowed one-pot synthesis, and the sulfide group is likely to be a potent biocide. The biocidal activities of the Au-DHT bioconjugates were compared to those of pure DHT and of the commercially available antibiotic streptomycin, the fungicide dithane-M45, and the insecticide permethrin.

## Results and discussion

### Biosynthesis and characterization of Au NPs

**Figure 1:**
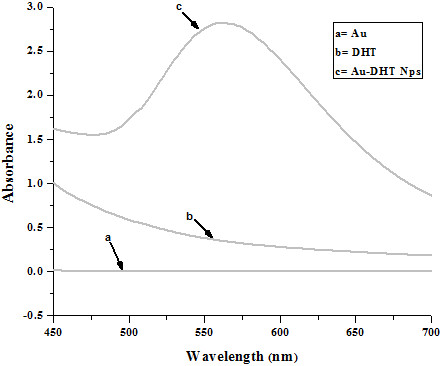
UV-vis spectra of Au, DHT and Au-DHT NPs.

When DHT was added to a HAuCl_4_ solution, the color of the liquid rapidly changed from light yellow to brown, indicating reduction of the Au ions and formation of Au nanoparticles. UV-Vis spectra of the suspensions are reported in Figure 1 and they show an absorption maximum at 562 nm.

Typically, water suspensions of Au nanoparticles have an absorbance maximum between about 520 and 530 nm [6]. A red shift in the plasmon absorption of Au nanoparticles can be induced by changes in the surface chemistry or by nanoparticle aggregation. For example, it is known that sulfur-containing capping agents can change the electron density of Au and therefore shift the position of the plasmon absorption. However, the red-shift induced by thiols is typically on the order of 5-10 nm, and it can account only very partially for our results. Spectral shifts of the magnitude observed in our experiments typically are due to aggregation or to a non-spherical shape of the nanoparticles [7].

**Figure 2:**
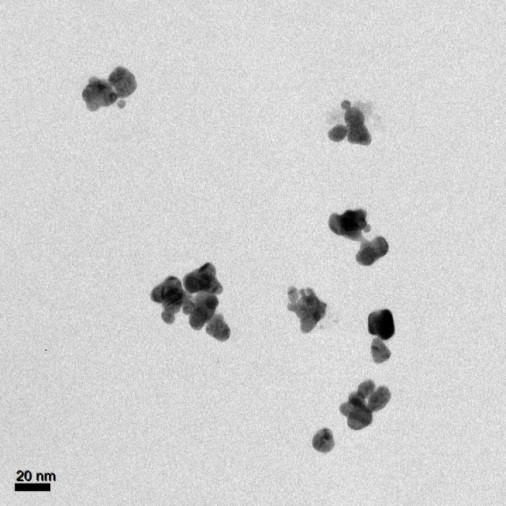
Bright field TEM image of Au-DHT bioconjugates.

The nanoparticles were thus characterized with TEM. Bright field images are reported in Figure 2 and they show that the nanoparticles had a non-spherical shape. The morphology of the nanoparticles suggested that nanoparticles of about 10 nm in diameter coalesced into larger aggregates with an irregular shape and a size on the order of 30 nm. For this type of irregularly shaped nanoparticles, a strongly red-shifted absorption is expected.

**Figure 3:**
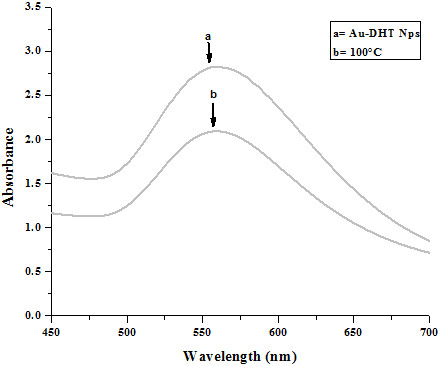
Effect of heat on stability of Au-DHT NPs: after 24 h.

**Figure 4:**
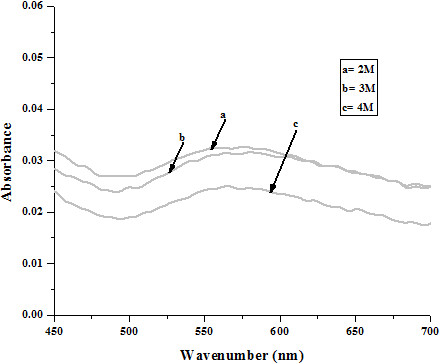
Effect of different salt concentrations (NaCl) on stability of Au-DHT NPs: after 24 h.

**Figure 5:**
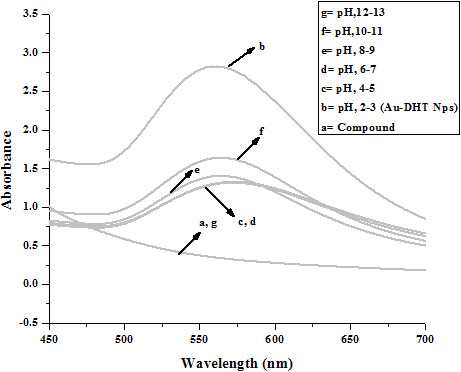
Effect of pH on stability of Au-DHT NPs: after 24 h.

We attribute the formation of aggregates to the presence of two sulfur atoms per DHT molecule, which might have acted as bridges between NPs. Whatever the origin of the aggregation; it did not compromise the stability of the suspensions, which could be kept for days at room temperature without any noticeable shift in their absorption. Precipitation was also not observed. The suspensions were also found to be stable under quite severe environmental modifications. Figure 3 reports absorption spectra of suspensions kept at elevated temperatures for 24 hours. The plasmon peak did not sift, and no precipitation was observed. The reduced absorbance is in agreement with previously reported results by the group of El-Sayed. They explained it on the basis of dominant electronic dephasing mechanism which involves electron-electron interactions as higher electronic temperatures do not only lead to a faster electron-electron scattering rate but should also increase the electron-surface and electron-defect scattering. The velocity of an electron depends on its state energy and hence on the temperature. It rises for higher excited electronic states. As an increase in the velocity of the electrons leads to a larger damping constant and therefore to a faster dephasing and hence resulting in the reduction of absorbance of plasmon band [8]. Variations of salt concentration and pH also did not affect the absorption spectra and the stability of the suspensions, as shown in Figures 4 and 5, respectively.

Figure 4 shows the effect of various concentrations of sodium chloride aqueous solution on surface plasmon peak of Au NPs. The result shows that higher concentration of NaCl resulted in a decrease in *A*_*max*_*.* The full width at half maximum (FWHM) also increases and thereby decreasing the stability of nanoparticles. This rapid decrease in absorbance of Au NPs containing NaCl is attributed to the aggregation effect promoted by Cl^-1^ ions. From these observations it was concluded that at higher concentration of sodium chloride, however, aggregation turned out to be dominant. As for as long term stability is concerned, Au NPs are much more stable in neat water that those in NaCl solution [9].

**Figure 6:**
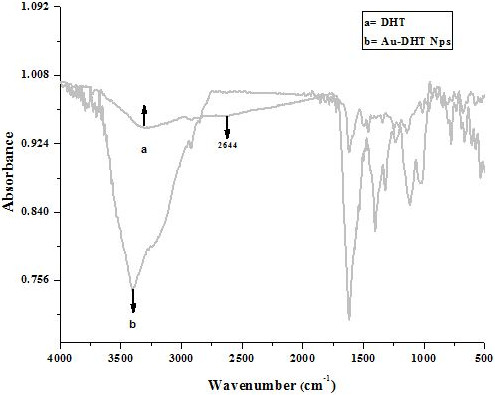
FTIR spectra of DHT and DHT-Au NPs.

Conjugation of DHT to Au was confirmed by FT-IR spectroscopy, which is reported in Figure 6. A peak at 2644 cm^-1^ attributed to thiol was measured in DHT but not in the conjugates, consistent with sulphur binding to the surface of the Au NPs.

**Figure 7:**
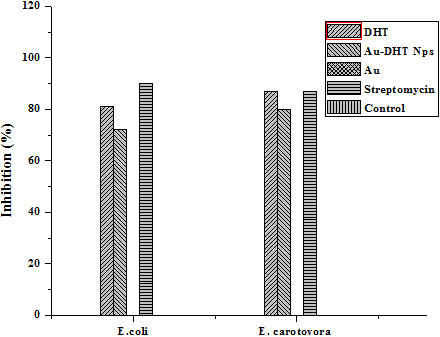
**Antibacterial activity (percentage inhibition) of DHT, Au-DHT NPs and Au (pure gold).** Standard drug: Streptomycin, Control: Bacteria only.

**Figure 8:**
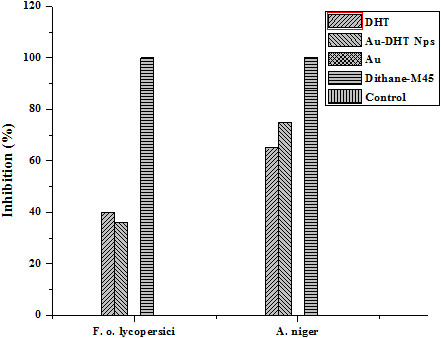
**Antifungal activity (percentage inhibition) of DHT, Au-DHT NPs and Au (pure gold).** Standard fungicide: Dithane-M45, Control: Fungi only.

**Figure 9:**
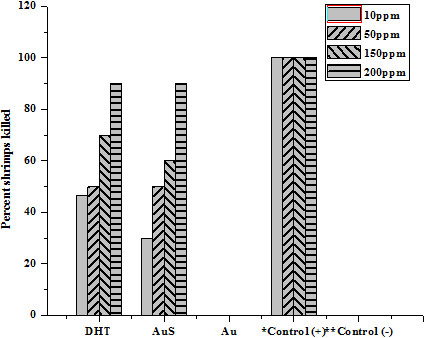
**Brine shrimp lethality of DHT, Au-DHT NPs and Au (pure gold).** * toposide at 7.4625 μg/ml and **methanol were used as the standard drug and used as negative control, respectively.

**Figure 10:**
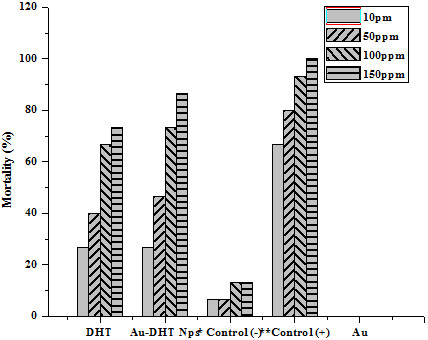
**Insecticidal activity of DHT, Au-DHT NPs and Au (pure gold).** *Permethrin at 235.9 mg/cm^2^ and **methanol were used as standard drug in the positive control and negative control respectively.

To measure the amount of conjugated DHT, Au nanoparticles were centrifuged out of the suspensions. The supernatant was freeze-dried and the residue weighed. These results indicate that the conjugates contained about 7% by weight of DHT. The conjugates were then tested as antibacterial, antifungal, insecticidal and cytotoxic agents and compared with pure DHT and with commercial biocides. In the tests, cell cultures were contacted with the same weight of Au-DHT conjugates, pure DHT and streptomycin (in the antibacterial test), Dithane-M45 (in the antifungal test), Etoposide (in the brine shrimp lethality bioassay), Permethrin (in the Insecticidal). The antibacterial activities of samples DHT and Au-DHT NPs are shown in Figure 7. Both DHT and Au-DHT NPs showed profound inhibition in the growth of the two bacterial species. This inhibition in the growth of bacterial species was almost similar as with the commercially available drug, streptomycin. The antifungal activity of sample DHT and Au-DHT NPs against two fungal species *Fusarium* oxysporum f. sp. *lycopersici* race 1 CUI and *Aspergillus niger* are shown in Figure 8. Au-DHT NPs showed best antifungal activity and resulted 75% inhibition in the growth of *Aspergillus niger*. DHT and Au-DHT NPs showed comparatively same antifungal activity against *Fusarium* oxysporum f. sp. *lycopersici* race 1 CUI. In case of Brine shrimp lethality bioassay both DHT and Au-DHT NPs showed low cytotoxic activity against the experimental shrimps in comparison to standard drug (Figure 9). However, the% mortality of brine shrimp larvae at 50 and 200 ppm of DHT and Au-DHT NPs was same. The highest dose 200 ppm of both the samples resulted in highest mortality (90%) of shrimp’s larvae. The dithioic acid functional group was also screened for insecticidal activity in pure DHT and its Au NPs, using impregnated filter paper method (Tabassum *et al*., 1997), against *Myzus Persicae*. The positive and negative controls showed 100% and 0% mortality, respectively. The effect of different doses of DHT and Au-DHT NPs are shown in Figure 10. Au-DHT NPs was more effective than DHT at all doses except at 10 ppm. Pure Au was inactive in all biocidal activities so these tests consistently indicated that Au-DHT nanoparticles had a biocidal activity which was comparable to that of pure DHT and the commercially available products. Most importantly, on a per weight base, our conjugates contained about 7% biocide. This indicates that conjugation enhanced the bioactivity of DHT by more than 14 times. At this stage, we do not have a clear idea of the mechanism underlying biocidal activity amplification. The results clearly indicate, however, that nanoparticle conjugation could be used to minimize the amount of biocide molecules, thereby delaying development of resistant strains and minimizing the environmental effects of biocides.

## Conclusions

In summary, in all cases, the Au-DHT conjugates were more efficient biocides than pure DHT, and had a comparable activity to that of the commercial products. On a per weight basis, our Au-DHT bioconjugates are also more efficient than commercial products, since they require about 14 times less biocide to reach the same activity. Our results are of great relevance. From the applied viewpoint, we notice that Au-DHT conjugates are very efficient against aphid vectors, a category of crop pests for which currently no potent chemicals or insecticides exist. *Myzus persicae* (Sulzer), a green peach aphid, is very damaging to peaches and nectarines and is a vector of virus diseases in crucifers, potatoes, and other crops. In Australia, this species is controlled mainly using organophosphate insecticides such as demeton-S-methyl and dimethoate. Resistance of *M. persicae* to these insecticides has been recorded from a number of countries [10] and was suspected in Australia [11]. From the basic science viewpoint, our results show that biocidal potency is amplified by conjugation to an otherwise biologically inert metal such as Au.

## Methods

### Materials and instruments

Tetrachloroauric (III) acid tri hydrate (HAuCl_4_), resorcinol, carbon disulfide (CS_2_), potassium hydroxide (KOH), absolute ethanol (EtOH) were purchased from Merck. A digital pH meter model 510 (Oakton, Eutech) equipped with a glass working electrode and a reference Ag/AgCl electrode was employed for pH measurements. UV-vis spectra were recorded with a Shimadzu UV-240, Hitachi U-3200 spectrometer with a path length of 1 cm. FT-IR spectra were recorded with a Shimadzu IR-460 spectrophotometer. A 1:1 mixture of freeze-dried Au-DHT NPs and KBr was pressed into a pellet. Proton NMR spectrum was recorded on Bruker AV-300 MHz spectrometers using MeOH-d_4_ as a solvent and TMS as an internal standard. TEM images were taken with a Zeiss Libra transmission electron microscope (TEM) operated at 120 keV.

### Synthesis of 2,4-dihydroxybenzodithioic acid (DHT)

A mixture of resorcinol (0.01 mol, 1.86 g) in ethanol (25 mL) and KOH (2 M, 0.56 g) in 5 mL water were taken in a 50 mL RB flask and refluxed for 30 min. CS_2_ (0.01 mol, 0.76 g) was added drop wise and the mixture was refluxed further for 3 h. The solution was cooled to room temperature and acidified with small portions of HCl (2 M). The crude product was recrystallized from EtOH [5].

**Figure 11:**
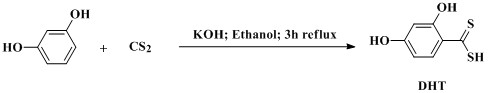
Synthesis of 2,4-dihdroxybenzene carbodithioic (DHT).

Yield: 82%, M.P.: 132–133°C. EIMS *m/z*: 186. ^1^H NMR (300 MHz, MeOH-d_4_): δ 8.421-8.391 (d, 1H, *J* = 9Hz, Aromatic), 6.839-6.801 (dd, 1H, *J* = 2.4Hz, *J* = 9Hz, Aromatic), 6.634-6.627 (d, 1H, *J* = 2.1Hz, Aromatic) (Figure 11).

### Synthesis of Au NPs

As DHT has low solubility in water, therefore a 10% mixture of methanol and water was used as a solvent for preparing 1 mM solution of DHT. 1 mM solution of tetrachloroauric (III) acid was prepared in water. 1 mL of 2,4-Dihdroxybenzene carbodithioic acid (1 mM) was added drop wise to 6 mL of tetrachloroauric (III) acid (1 mM). During addition, the color of the reaction mixture changed from yellow to dark brown. The resulting mixture was stirred for 3 h at room temperature. Solid Au-DHT NPs were collected by freeze drying.

### Bioassay of gold nanoparticles

#### Brine shrimp lethality bioassay

Cytotoxic activity of the samples was determined by Brine shrimps lethality bioassay [12]. The eggs of brine shrimp were collected from Department of Botany, University of Peshawar and hatched in a tank at room temperature with constant oxygen supply. After hatching for two days, the mature nauplii were produced and the eggs were placed in 3.8% sea salt solution. Various concentrations i.e. 10, 50, 100 and 200 ppm were made with respective solvent and were allowed to evaporate. Total of 10 larvae were added in each concentration and stored at room temperature. Control treatments were also run in parallel as reference to compare toxicity and LD_50_ values were calculated using probit analysis.

#### Antifungal activity

The antifungal activity was evaluated by the agar-well diffusion method [12]. The test sample (20 mg in DMSO) was uniformly dissolved in PDA medium on agar plate. Using sterilized borer mycelial plug (5 mm) of *Fusarium oxysporum* f. sp. *lycopersici* (FOL) and *Aspergillus niger* were fixed at the center of the agar plate. The positive control (Dithane-M45 fungicide) and negative control (media + DMSO) were also run in parallel for reference. The agar plate cultures were incubated at 25°C for 7 days and growth of the fungal strains were observed on daily basis. After incubation, the cultures were compared for reduction in radial colony growth of the fungus in negative, positive and test cultures. Percent growth Inhibition in radial colony growth was determined by using the formula,

$$I=\left(C-T\right)/C\times 100$$

I = Percentage of inhibition, C = Diameter of fungal colony in control, T = Diameter of the fungal colony in treatment.

#### Antibacterial activity

The antibacterial activity of samples was determined according to method described by Yin *et al.* (2010). For this study two gram-negative bacterial species were selected. *Escherichia coli* is a rod-shaped bacterium that is commonly found in the lower intestine of warm-blooded organisms [13]. *Erwinia carotovora* infects a variety of vegetables and plants including carrots, potatoes, cucumbers, onions, tomatoes, lettuce and ornamental plants like iris [14]. Each sample about 0.5 mg was taken in a test tube and added to 1 mL DMSO. In sterilized petridishes, 10 mL PDA was poured and bacterial inoculation was carried out by streaking to form different colonies. The disc of 5 mm was kept at the center of agar plate and 10 μL of sample was poured on disk. Positive and negative control was also taken and zone of inhibition was observed after 72 hours.

$$\mathrm{IE}\left(\%\right)=\mathrm{DC}-\mathrm{DS}/\mathrm{DC}-5\times 100$$

DC stands for diameter of control and 5 is the size of disc which was 5 millimeter.

#### Insecticidal activity

The insect specie *Myzus persicae* (green peach leaf aphid) was obtained from Entomology Department, Khyber Pakhtunkhwa Agricultural University Peshawar. The insecticidal activity was performed by previously reported method [15]. Permethrin (235.71 μg/cm^2^) and methanol were used as positive and negative controls, respectively.

## Abbreviations

DHT: 2-4, Dihydroxybenzene carbodithioic acid; EIMS: Electron impact mass spectrum; Au NPs: Gold nanoparticles; UV: Visible spectra; Ultra violet: Visible spectra; FWHM: Full width at half maximum; FTIR: Fourier transformed infrared; TEM: Transmission electron microscope.

## Competing interests

The authors state that they have no competing interests.

## Authors’ contributions

SSN, SSA, ZI, LF and AA performed the synthesis and as well as biological evaluations, MB and NUI helped with the analysis, MB and MRS wrote the manuscript and supervise the whole work. MRS conceived the idea. All authors read and approved the final manuscript.
